# Validity of a Wearable Digital Insole for Assessing Gait ON and OFF in Parkinson's Disease

**DOI:** 10.1002/acn3.70333

**Published:** 2026-02-24

**Authors:** Deborah A. Hall, Kimberly Kwei, Rolando J. Acosta, Bharatkumar Koyani, Erin Robertson, Pratyush Rai, Roland Barge, Emily Timm, Nicollette L. Purcell, Natasha Desai, Dhanesh Patel, Ana‐Maria Visoiu‐Knapp, Samuel Stuart, Rinol Alaj, Matthew F. Wipperman, Oren Levy, Joan A. O'Keefe

**Affiliations:** ^1^ Department of Neurological Sciences Rush University Medical Center Chicago Illinois USA; ^2^ Department of Neurology, Movement Disorders Columbia University Medical Center New York New York USA; ^3^ Regeneron Pharmaceuticals, Inc. Tarrytown New York USA; ^4^ Department of Anatomy & Cell Biology Rush University Medical Center Chicago Illinois USA; ^5^ Illinois College of Optometry Chicago Illinois USA

**Keywords:** digital insole, Parkinson's disease, wearables

## Abstract

**Objective:**

Gait impairment is a distinctive symptom of Parkinson's disease that negatively impact mobility. We assessed the validity of wearable digital insoles against a validated reference gait analysis system for measuring select gait characteristics in patients with Parkinson's disease.

**Methods:**

A comparative analysis between digital insoles (Moticon ReGo Insole) and the GAITRite system was conducted in patients with Parkinson's disease. Patients were assessed in both the OFF and ON medication states. Gait characteristics were measured simultaneously with both systems during two 10 m walk tests. Patients also completed a patient experience survey following the use of the digital insoles.

**Results:**

Overall, 21 patients with Parkinson's disease were included in the study. Analytical validation for gait cadence, speed, and stride length showed excellent agreement (intraclass correlation coefficients between 0.93–0.97) in both the OFF and ON states. Stance, swing, and double support times exhibited lower validity with moderate agreement (intraclass correlation coefficients from 0.48–0.57). Gait speed and stride length were significantly associated with scores on the Movement Disorders Society's Unified Parkinson's Disease Rating Scale (*p* = 0.0085 and 0.013, respectively). Mean differences in all parameters measured with the insoles, except cadence, were significantly different between OFF and ON states (*p* < 0.003). The majority of patients liked wearing the digital insoles and found them comfortable and user‐friendly.

**Interpretation:**

These findings support the validity of Moticon ReGo digital insoles for the assessment of several important gait characteristics in Parkinson's disease.

## 1 Introduction

Gait impairment and falls are associated with significant morbidity and mortality in patients with Parkinson's disease (PD) [1]. Gait impairment in patients with PD is less responsive to pharmacological intervention than other motor signs, therefore therapeutics that are able to decrease freezing, falls, and other gait abnormalities are critically needed. In the clinical setting, neurologists and other clinicians evaluate gait using standard, validated motor rating scales, such as the Movement Disorders Society's Unified Parkinson's Disease Rating Scale (MDS‐UPDRS) [2]. The scale allows assessment of the ON or OFF levodopa state, which is important for interpretation of the scores and adjustment of therapeutics in the clinical setting. The MDS‐UPDRS has some psychometric limitations due to floor effects and disordered thresholds, making it less useful in patients with early PD [3]. Scales specifically focused on gait and balance in PD perform suboptimally or have been evaluated insufficiently [4]. This has created a need not only for a well‐validated PD‐specific gait and balance rating scale, but also for quantitative digital measures that could be used to supplement existing scales. Novel technologies could also facilitate the measurement of more complex gait impairments, such as turning, difficulties in initiating and stopping movements and evaluation of gait in both the “OFF” and “ON” states, and could be used remotely in the home setting more frequently than clinician assessment [5].

Detailed, quantitative gait assessment has traditionally been performed in laboratory environments using sophisticated and expensive equipment operated by highly trained staff. These labs may include 3D motion capture systems, pressure‐sensitive walkways, and force plates, each of which can measure various aspects of the gait cycle and ground‐reaction forces [6]. Controlled laboratory environment studies testing PD patients in the ON or OFF levodopa state can produce results with low ecological validity. More recent technological advancements have allowed for the development of wearable technology, such as digital insoles or inertial measurement units (IMUs), which can automatically measure, calculate, and provide digital mobility outcomes to clinicians or researchers in near real time for a much lower cost. Developing validated digital insoles may allow this research to be conducted in the real world and overcome current limitations.

Digital insoles incorporating capacitive pressure measurement sensors combined with inertial accelerometer and gyroscope sensors have been used previously to measure gait in patients with PD [7, 8, 9, 10, 11, 12, 13]. Many of these devices offer superior accuracy in the detection of initial and final foot contact over IMUs alone due to the inclusion of plantar pressure force sensors [8, 11, 14]. Algorithms that process raw pressure and accelerometry data from digital insoles can now provide highly precise measures of spatiotemporal aspects of gait and the detection of gait events important in PD, including gait initiation, turning, and freezing [14, 15].

The purpose of this study was to compare a digital insole (Moticon ReGo) to the GAITRite gait measurement system, a validated reference method, by capturing spatial and temporal gait characteristics in patients with PD. While not the first, this is one of the early studies to address these questions in a controlled experimental setting. Additionally, gait characteristics were compared in the ON versus OFF medication state; their association with severity of PD motor signs as measured by the MDS‐UPDRS Part III motor score was also examined.

## 2 Methods

### 2.1 Study Design

This was a prospective, open‐label, observational, cross‐sectional, investigator‐initiated study conducted at two sites: Rush University Medical Center and Columbia University Medical Center. The protocol was written by the study teams at the two sites in collaboration with Regeneron Pharmaceuticals Inc. Sites obtained local institutional review board approval prior to patient enrollment (Rush ORA#22011806). Inclusion criteria included clinical diagnosis of PD by a movement disorder neurologist (D.A.H. or K.K.) [16], age 40–80 years, taking levodopa and/or other dopaminergic medications, willing to undergo testing in the OFF and ON state, ambulatory without an assistive device in the OFF state, able to complete questionnaires, and able to consent. Patients over 70 were originally excluded due to higher likelihood of non‐PD related balance difficulties over age 70, however, patients between 70 and 80 were added to the inclusion criteria when recruitment was slower than expected. Exclusion criteria included dementia by clinical history, significant leg or back arthritis, signs/symptoms of atypical parkinsonism, previous stroke, or recent orthopedic surgery. Participants were recruited by their movement disorder neurologist and/or clinical research coordinators at their clinic visits. Safety monitoring was performed by the site principal investigators (D.A.H., K.K.).

### 2.2 Demographic and Clinical Assessments

After informed consent, demographic information, medical/surgical history, and concomitant medications were collected. Patients were asked to wear comfortable gym shoes that allowed insertion of the insoles appropriately and comfortably. Each patient had a physical examination, including vital signs. For motor testing, patients were first assessed in the OFF state in the morning, defined as being off dopaminergic medications for at least 12 h. They were reassessed 30–60 min after taking the first dose of levodopa or any other dopaminergic medications once participants confirmed they were in their typical ON state. The same site investigator performed and scored the MDS‐UPDRS Part III in the OFF and ON states.

### 2.3 Digital Insoles

**FIGURE 1:**
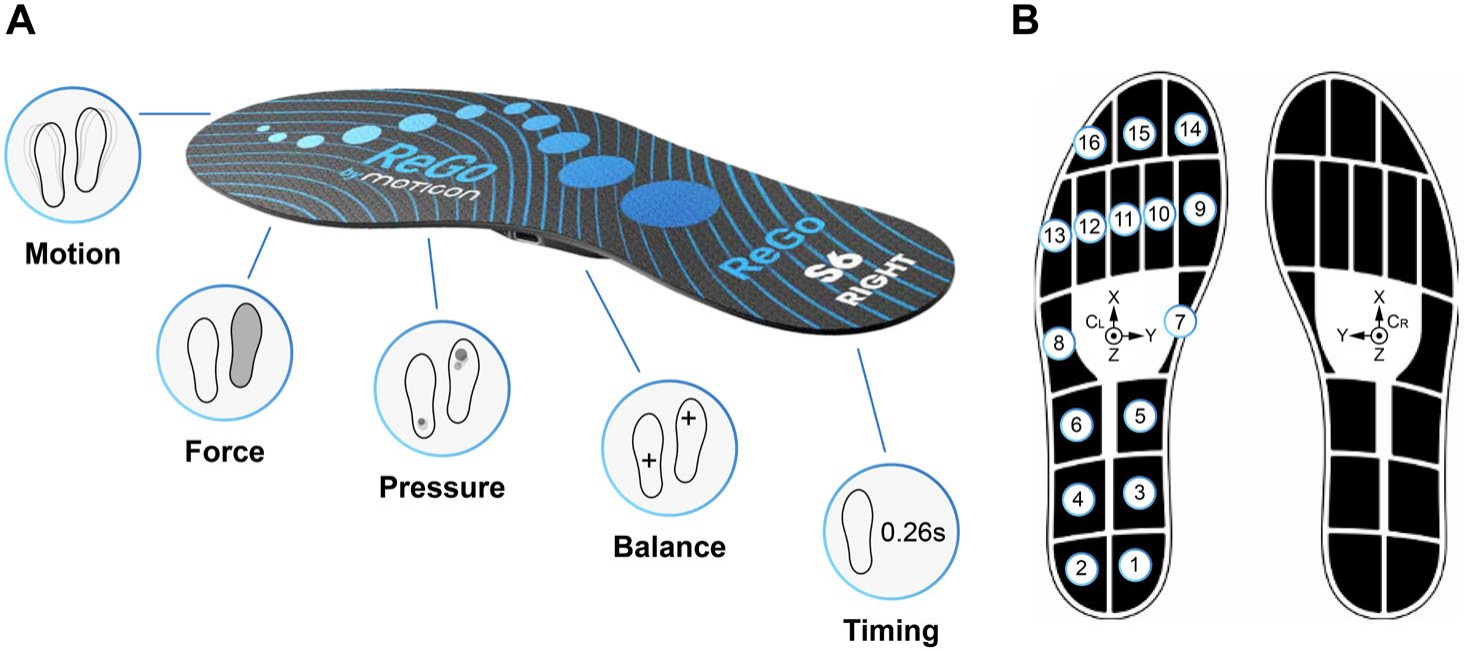
Moticon ReGo insole. (A) Illustration of the Moticon ReGo Insole, (B) the ReGo Insole with 16 capacitive pressure sensors with an integrated inertial measurement unit (a 3D accelerometer and a 3D gyroscope) for measuring both plantar pressure and motion. Figures used and modified with permission from Moticon ReGo AG.

During the walking trials, the digital insole (Moticon ReGo Insole) device was tested with the GAITRite instrumented walkway (6 m × 0.6 m; version 4.89K9c; sampling frequency of 120 Hz; Figure 1). Digital insole pressure and accelerometry data were collected at the rate of 100 Hz, stored on the insole's onboard SD memory card, and then transferred via Bluetooth to a smartphone running the Moticon ReGo app before being uploaded to the cloud. The ReGo Cloud uses the BaaS (Backend as a Service) framework, Google Firebase. In particular the following components: (1) Firestore: used for ReGo Test results, user profiles, user settings (e.g., schedules) and database location in EU; (2) Cloud storage: used for sensor data, backup; database across multiple locations in EU; (3) Firebase Auth: used for user authentication and login credentials (username, password, PIN), database location in the United States; (4) Cloud functions: used for execution of backend functionalities and database location in the European Union.

Gait characteristics were derived from the digital insole manufacturer's ReGo software (Moticon ReGo AG, Munich, Germany). The insoles were worn in both shoes (after removing the patients' standard insoles) during system calibration and a brief walking warm‐up, which allowed the devices to acclimate to body temperature. This was performed because large fluctuations in temperature and humidity can lead to measurement drift and cause frequent recalibrations. The digital insoles were calibrated on the ReGo mobile app to the patients' bodyweight, per the manufacturer's guidelines. The calibration included patients walking slowly for ~40 s, standing still for 10 s, and performing 10 s each of anterior–posterior and lateral weight shifts. After calibration, successful zeroing was confirmed by visualizing zero total force on either insole of a raised leg during a unilateral stance.

For the gait assessments, patients were asked to perform a 10 m walk task, defined as: starting from a standing position, walking freely at a comfortable self‐selected speed for 10 m, turning around (off the GAITRite walkway), and walking back 10 m (returning to the starting point). Patients performed this task twice in both the OFF and ON states on the GAITRite system, while wearing the Moticon ReGo wearable insoles, in order to simultaneously measure gait with both devices. Patients could rest as needed between each trial of the walking test.

### 2.4 Outcome Measures

The aims of the study were as follows. First, to evaluate the concordance between the digital insole gait characteristics and GAITRite parameters, the chosen reference system. The second aim was to assess the ability of the digital insole to detect change from the OFF state (before levodopa or other dopaminergic medication administration) to the ON state (after medication administration). The third aim was to evaluate the relationship between the digital insole gait characteristics and MDS‐UPDRS Part III motor scores. Primary endpoints included: (1) concordance of the same gait characteristics derived from the digital insoles and GAITRite; (2) change in gait from OFF state to ON state, as measured by the digital insoles; and (3) association between the gait characteristics derived from digital insoles and motor score of the MDS‐UPDRS Part III. For GAITRite, raw footfall data was visually inspected and partial footfalls were manually removed. Once processing was completed, the data was exported in a CSV file. Gait measures derived from the GAITRite walkway and digital insoles were processed using manufacturer‐provided software, and averaged values over the 10 m walk test were exported for further analysis. The following gait characteristics derived from both devices were analyzed: gait speed (m/s); stride length (m); cadence (steps per minute); swing time (percentage gait cycle time [GCT]); stance support time (percentage GCT); and double support time (percentage GCT).

### 2.5 Patient‐Reported Outcomes

Immediately following the gait test, patients were asked to complete an electronic patient experience questionnaire deployed on the SurveyMonkey platform (SurveyMonkey Inc., San Mateo, CA, USA) to capture patient feedback on the comfort, satisfaction, willingness to use at home, and acceptable test frequency (the survey is provided in Table S1). The survey contained questions on a Likert scale with low scores indicating high ease of use, satisfaction, comfort, etc. Open‐ended and yes/no questions were also included in the questionnaire to capture any potential issues or concerns.

### 2.6 Statistical Analyses

Patient demographic and clinical data were collected and stored in a REDCap database, before being de‐identified and transferred to Regeneron Pharmaceuticals Inc. Summary statistics were completed for patient demographics and characteristics. Right and left gait characteristics were averaged (e.g., swing time with the left foot and swing time with the right foot). Corresponding gait characteristics were measured using the same units. Summary statistics were provided for each gait characteristic by measuring system (ReGo digital insole or GAITRite) and patient state (ON or OFF). Concordance between the two systems was assessed using the absolute agreement intraclass correlation coefficients (ICC_2,1_), the mean differences with corresponding 95% confidence intervals (CIs), limits of agreement (LoAs), and Pearson correlation coefficients. A mixed‐effects linear model to assess characteristic‐specific mean differences between measuring systems was used. The mean bias (mean difference between the two measuring systems) and corresponding 95% CIs were extracted from this model using Bland and Altman analysis [17]. The 95% LoAs were calculated as the bias plus or minus two standard deviations of the difference between the systems. The ICC_2,1_ findings were interpreted as follows: excellent (≥ 0.90); good (0.75–0.89); moderate (0.50–0.74); and poor (< 0.50) [18, 19].

To examine the responsiveness of the digital insoles to dopaminergic therapy, the average change in gait characteristics between the ON and OFF states was performed using a linear mixed‐effects regression model that adjusted for the MDS‐UPDRS Part III score in the OFF state. Associations between the ReGo insole‐derived gait characteristics and the MDS‐UPDRS Part III score were assessed via regression analysis, controlling for medication state and enrollment site. The same analyses were performed for the GAITRite‐derived gait characteristics. Given the pilot nature of this study, the statistics were not adjusted for multiple comparisons. All data analyses were conducted in R version 4.3.0 (R Core Team, Vienna, Austria).

For the patient‐reported outcome survey, direct counts of each option in the Likert scales were obtained and converted to percentages. Comments were manually codified and thematic and sentiment analysis conducted via counts and percentages.

For sample size calculation, the mean and standard deviation of gait characteristics from a reference population (*n* = 119) of patients with early PD [20] were used to estimate a sample size to reach significance for the primary outcome measures. In the Rehman study [20], gait was quantified using an instrumented GAITRite walkway, from which 16 gait characteristics were derived and assessed. The observed means and standard deviations from this study, along with the expected 95% CI half‐width at the interim supported a sample size of 20 patients, which would provide a 95% CI for the following measures: pace; rhythm; variability; asymmetry; and postural control.

## 3 Results

Twenty‐one patients with PD were recruited and attended a single visit at either Columbia University Medical Center or RUSH University Medical Center to complete clinical and gait assessments. Patient demographic and clinical characteristics are shown in Table 1. All participants were able to do study activities in ON and OFF states. The time participants were in their OFF state ranged from 12 to 17 h. The first dose of the day was taken at various times but ranged from 8:30 am to 1 pm. The following medications were taken by the participants: carbidopa/levodopa (*n* = 17), carbidopa/levodopa extended release (*n* = 9), Rytary (*n* = 1), Inbrija (*n* = 1), amantadine or amantadine extended release (*n* = 10), selegiline or rasagiline (*n* = 7), rotigotine (*n* = 2), pramipexole (*n* = 4), entacapone or opicapone (*n* = 4), trihexyphenidyl (*n* = 1). Four participants took only carbidopa/levodopa extended release as the first dose of the day. The average levodopa equivalent daily dose (LEDD) for participants was 1033.1 ± 520.2 mg.

**TABLE 1:** Patient demographics.

Demographic	OFF state	ON state
Age, years, mean (SD)	64.2 (10.5)	
Sex, *n* (%)		
Female	10 (47.6)	
Male	11 (52.4)	
Disease duration, years, mean (SD)	9.57 (5.70)	
Levodopa equivalent daily dose mean (SD)	1033.1 ± 520.2	
Hoehn & Yahr scale stage, *n* (%)		
2	17 (81.0)	19 (90.5)
3	4 (19.0)	2 (9.5)
MDS‐UPDRS Part III score, mean (SD)	43.4 (18.3)	24.3 (11.4)

Abbreviations: MDS‐UPDRS, Movement Disorders Society's Unified Parkinson's Disease Rating Scale; SD, standard deviation.

**FIGURE 2:**
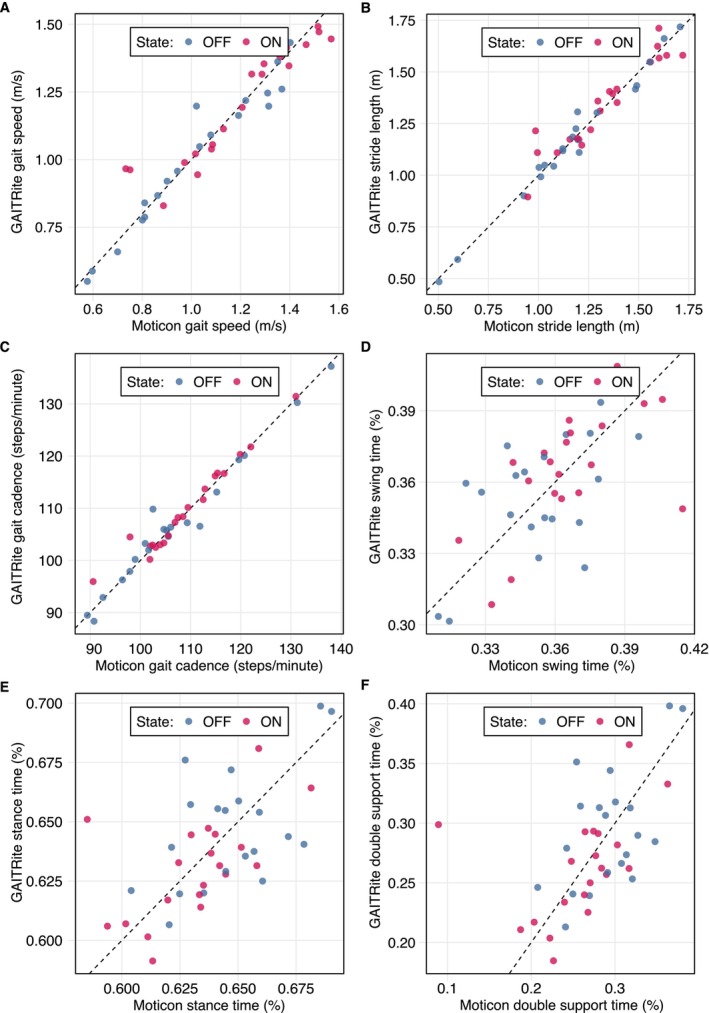
Scatter plots of gait characteristics between the ReGo insoles and the GAITRite in both the OFF and ON states. (A) Gait speed, (B) Stride length, (C) Gait cadence, (D) Swing time, (E) Stance time, (F) Double support time.

**FIGURE 3:**
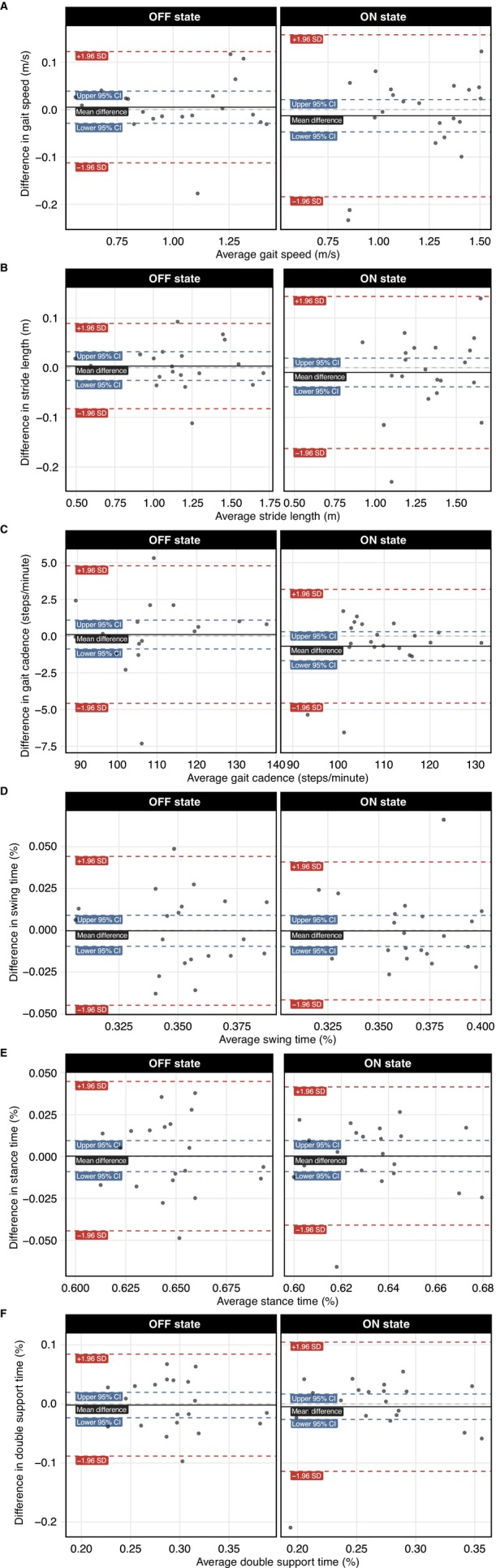
Bland–Altman plots for gait characteristics from the ReGo insoles and the GAITRite system in both the OFF and ON states. The solid gray line represents the mean difference between devices with the upper and lower 95% CI in the dashed blue line. The dashed red lines represent the 95% LoAs between the devices. CI, confidence interval; LoA, limit of agreement. (A) Gait speed, (B) Stride length, (C) Gait cadence, (D) Swing time, (E) Stance time, (F) Double support time.

**FIGURE 4:**
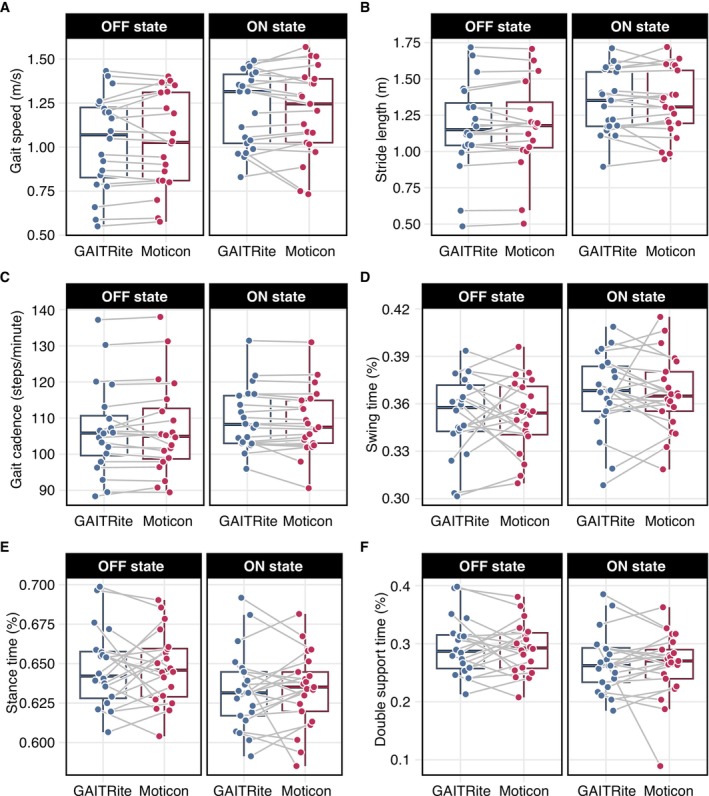
Gait characteristics collected by both measuring systems. (A) Box plot of gait speed by measuring platform. Blue data points are measurements taken with the GAITRite mat, and pink data points are measurements taken with the digital insoles (Moticon, ReGo). Individual‐specific observations are connected with gray lines. OFF and ON state measurements are presented in different facets and labeled at the top of the figure. Panels (B–F) illustrate stride length, gait cadence, swing time, stance time, and double support time, respectively.

For the first aim, six gait characteristics were simultaneously measured by the digital insoles and GAITRite (Table 2; Figures 2, 3, 4). The mean difference between the measuring platforms was small for all of the six gait characteristics studied and the corresponding 95% CI overlapped with zero, showing that these differences were not statistically significant. Mean differences between the measuring systems were not affected by patient ON or OFF state. The agreement between the two systems for gait cadence, gait speed, and stride length was excellent, with ICC_2,1_ values over 0.93. Swing (ON and OFF), stance (ON and OFF), and double support time (OFF) showed moderate agreement between the systems. Of the three, double support time in the ON state showed the worst agreement, with an ICC_2,1_ of 0.48 (95% CI: 0.06–0.76; Table 2). The Pearson's correlation coefficients mirrored the agreement findings, where gait cadence, gait speed, and stride length showed strong correlations between measuring systems (*r* = 0.94–0.99) and swing, stance, and double support time had weaker correlations (*r* = 0.48–0.64).

**TABLE 2:** Comparison of the digital insole (Moticon, ReGo) to GAITRite.

Characteristic	Moticon	GAITRite	Mean difference	LoA	ICC	*r*
Temporal – OFF state						
Speed, m/s (95% CI)	1.03 (0.92, 1.15)	1.03 (0.91, 1.14)	0 (−0.03, 0.04)	−0.12 to 0.12	0.98 (0.94, 0.99)	0.98
Stride length, m (95% CI)	1.17 (1.04, 1.31)	1.17 (1.03, 1.31)	0 (−0.03, 0.03)	−0.09 to 0.09	0.99 (0.98 to 1.00)	0.99
Cadence, steps per minute (95% CI)	106.94 (100.73, 113.14)	106.83 (100.63, 113.04)	0.1 (−0.88, 1.09)	−4.58 to 4.78	0.98 (0.96, 0.99)	0.98
Temporal – ON state						
Speed, m/s, (95% CI)	1.21 (1.11, 1.31)	1.22 (1.12, 1.32)	−0.01 (−0.05, 0.02)	−0.18 to 0.16	0.93 (0.84, 0.97)	0.94
Stride length, m (95% CI)	1.33 (1.23, 1.43)	1.34 (1.24, 1.44)	−0.01 (−0.04, 0.02)	−0.16 to 0.14	0.94 (0.85, 0.98)	0.94
Cadence, steps per minute, (95% CI)	109.29 (105.39, 113.18)	109.98 (106.08, 113.87)	−0.69 (−1.67, 0.3)	−4.56 to 3.18	0.97 (0.93, 0.99)	0.98
Spatial – OFF state						
Swing time, %GCT (95% CI)	0.35 (0.34, 0.36)	0.35 (0.34, 0.36)	0 (−0.01, 0.01)	−0.04 to 0.04	0.57 (0.17, 0.8)	0.56
Stance time, %GCT (95% CI)	0.65 (0.64, 0.66)	0.65 (0.64, 0.66)	0 (−0.01, 0.01)	−0.04 to 0.04	0.56 (0.16, 0.8)	0.55
Double support time, %GCT (95% CI)	0.29 (0.27, 0.32)	0.29 (0.27, 0.32)	0 (−0.02, 0.02)	−0.09 to 0.09	0.58 (0.18, 0.81)	0.57
Spatial – ON state						
Swing time, %GCT (95% CI)	0.37 (0.35, 0.38)	0.37 (0.36, 0.38)	0 (−0.01, 0.01)	−0.04 to 0.04	0.65 (0.29, 0.84)	0.64
Stance time, %GCT (95% CI)	0.63 (0.62, 0.65)	0.63 (0.62, 0.64)	0 (−0.01, 0.01)	−0.04 to 0.04	0.64 (0.29, 0.84)	0.63
Double support time, %GCT (95% CI)	0.26 (0.24, 0.29)	0.27 (0.24, 0.29)	0 (−0.03, 0.02)	−0.11 to 0.11	0.48 (0.06, 0.76)	0.48

Abbreviations: CI, confidence interval; ICC, intraclass correlation coefficient; LoA, limit of agreement.

For the second aim, each of the six gait characteristics was evaluated in the ON and OFF states (Table 3). With the exception of gait cadence, all gait characteristics as measured by the ReGo digital insole showed statistically significant mean differences between the ON and OFF states, with Cohen's d values ranging from 0.55 to 0.64 in absolute value. Gait speed and stride length increased 0.17 m/s and 0.15 m, respectively, from OFF to ON. Swing time increased 0.01% gait cycle time (GCT), and stance and double support time decreased 0.01% and 0.03% GCT, respectively. For the third aim, the association between the MDS‐UPDRS Part III and each gait characteristic was assessed (Table 4, Figure S1). When measured by the digital insoles, gait speed and stride length showed statistically significant associations with the MDS‐UPDRS Part III, adjusting for ON or OFF state. No other gait characteristics were significantly associated with the MDS‐UPDRS Part III. When measured by GAITRite, the results for gait cadence, gait speed, and stride length were similar.

**TABLE 3:** Change in gait characteristics between the ON and OFF state for both the Moticon ReGo insole and GAITRite.

Characteristic	Mean difference (95% CI)	Cohen's *d* (95% CI)	*p*
Moticon			
Speed, m/s	0.17 (0.08, 0.27)	0.64 (0.30, 0.98)	0.0002
Stride length, m	0.15 (0.06, 0.25)	0.55 (0.21, 0.89)	0.0012
Cadence, steps per minute	2.35 (−1.68, 6.38)	0.21 (−0.15, 0.58)	0.2431
Swing time, %GCT	0.01 (0.01, 0.02)	0.55 (0.25, 0.86)	0.0003
Stance time, %GCT	−0.01 (−0.02, −0.01)	−0.55 (−0.86, −0.25)	0.0003
Double support time, %GCT	−0.03 (−0.05, −0.01)	−0.55 (−0.90, −0.21)	0.0015
GAITRite			
Speed, m/s	0.19 (0.10, 0.28)	0.73 (0.40, 1.07)	< 0.0001
Stride length, m	0.17 (0.08, 0.26)	0.60 (0.27, 0.93)	0.0002
Cadence, steps per minute	3.15 (−0.82, 7.11)	0.29 (−0.08, 0.66)	0.1126
Swing time, %GCT	0.01 (0.01, 0.02)	0.52 (0.30, 0.74)	< 0.0001
Stance time, %GCT	−0.01 (−0.02, −0.01)	−0.52 (−0.74, −0.30)	< 0.0001
Double support time, %GCT	−0.03 (−0.04, −0.02)	−0.51 (−0.73, −0.29)	< 0.0001

Abbreviation: CI, confidence interval.

**TABLE 4:** Associations between each gait characteristic and the MDS‐UPDRS motor score.

Characteristic	Standardized association with motor score (95% CI)	Association with motor score (95% CI)	*p*
Moticon			
Speed, m/s	−0.12 (−0.20, −0.03)	−0.37 (−0.56, −0.11)	0.0085
Stride length, m	−0.11 (−0.19, −0.02)	−0.34 (−0.53, −0.08)	0.0130
Cadence, steps per minute	0.01 (−0.08, 0.11)	0.00 (−0.01, 0.01)	0.8317
Swing time, %GCT	0.00 (−0.11, 0.13)	0.16 (−0.99, 134.03)	0.9501
Stance time, %GCT	−0.00 (−0.11, 0.12)	−0.14 (−0.99, 99.28)	0.9501
Double support time, %GCT	−0.02 (−0.11, 0.09)	−0.29 (−0.90, 3.87)	0.7192
GAITRite			
Speed, m/s	−0.11 (−0.20, −0.02)	−0.37 (−0.57, −0.08)	0.0141
Stride length, m	−0.10 (−0.18, −0.01)	−0.32 (−0.52, −0.03)	0.0282
Cadence, steps per minute	0.01 (−0.08, 0.10)	0.00 (−0.01, 0.01)	0.9115
Swing time, %GCT	−0.09 (−0.20, 0.02)	−0.98 (−1.00, 1.26)	0.0985
Stance time, %GCT	0.10 (−0.02, 0.25)	46.28 (−0.57, 5156.38)	0.1002
Double support time, %GCT	0.11 (−0.02, 0.25)	6.32 (−0.30, 75.10)	0.0891

Abbreviations: CI, confidence interval; MDS‐UPDRS, Movement Disorders Society's Unified Parkinson's disease rating scale.

The patient experience survey (*n* = 21) results demonstrated that 86% of patients were either comfortable or very comfortable with wearing the insoles. Most patients (90%) reported that the digital insoles were easy to use, and 67% of patients reported that they were easy to adjust. When asked about overall satisfaction, 95% were either satisfied or very satisfied, and 72% of patients would recommend the insoles to others. When asked if they would be willing to use the digital insoles at home to complete the walking task more frequently, 76% stated that they would be willing or very willing and 50% responded that daily testing would be the most frequent assessment that they would find acceptable (33% reported that weekly testing would acceptable, 11% reported that every 2 weeks would be acceptable and 6% reported that monthly testing would be acceptable). Finally, sentiment and thematic analysis of the open‐ended questions showed that 44% were positively themed (e.g., comfortable, liked, and comfortable after adjustment), 38% were neutral (e.g., had to wear gym shoes, no cramping) and 18% were negatively themed (e.g., too tight, hard to install, height of heels, slightly warm in arch).

## 4 Discussion

Our results show that the digital insole (Moticon ReGo) is a valid device in ambulatory patients with PD to measure gait speed, stride length, and cadence. However, validation results for other metrics of the gait phase cycle were less than optimal. These results are consistent with a prior study looking at a similar digital insole (FeetMe) that showed high ICC_2,1_ (0.92–0.99) in mean stride velocity, cadence, stride length, and step, stride, and stance time but lower ICC_2,1_ for swing and double support time (0.61–0.73, respectively) compared to the GAITRite system [8]. In this prior study patients with PD were similar in age to our study; however, there were more men, the ON/OFF state was not specified, and they were earlier in the disease course. In another study, the same digital insole (Moticon ReGo) was compared to an IMU sensor system which consisted of five wearable devices as part of the PDMonitor medical device (PD Neurotechnology, London, UK) [10]. The IMU sensors showed high ICC_2,1_ (> 90) with several gait features, including cadence, gait cycle duration (right and left), single support (right and left), stance phase (right and left), and number of steps. Recently, gait metrics derived from an OpenGo Moticon sensor insole system were compared with those from the Protokinetics Zeno walkway in 12 patients with PD [7]. ICC_2,1_ were not computed, but Pearson's r demonstrated high correlations for gait speed, stride length, cadence, stance, swing, and double support time (*r* = 0.88–0.98). Higher agreement for gait phase cycle parameters compared to our study is unclear, but may have been because of differences in the proprietary Moticon OpenGo and ReGo algorithms. Several additional studies have shown the utility of digital insoles to measure freezing of gait [9, 11, 12, 13]. These studies confirm that digital insoles can reliably capture not only specific gait characteristics outside of a gait lab, but also characteristics unique to the PD patient population.

Reduced gait speed is associated with disability, frailty, falls, and reduced quality of life in individuals in the PD community [21], and most importantly is associated with increased mortality [22]. Gait speed and stride length (both of which are in the gait pace domain) are reduced in patients with PD [23], making them important outcome measures for interventions designed to improve gait bradykinesia. Gait cadence (a temporal parameter in the gait rhythm domain) is frequently abnormal in patients with PD, and together with speed and stride length, it is used to determine progression in rehabilitation [24]. Thus, these three gait characteristics of the wearable ReGo insole, which demonstrated excellent validity in this study compared to the reference system, offer important quantitative measures for gait interventions in patients with PD.

The gait characteristics that measure percentages of the gait phase cycle (swing, stance, and double support time) showed moderate agreement between the two measurement systems in both the OFF and ON states (except double support in the ON state). The digital insole in this study also captured differences in the ON and OFF states in patients with PD (except cadence), showing expected responses to dopaminergic state [10, 25, 26, 27, 28]. Gait cadence is influenced by many factors, especially the length of the walking bout [29]. The walking bouts used in this study were very short at 10 m (with actual quantitative measurement of 6 m). Dopaminergic medications might improve cadence over longer bouts of walking as bradykinesia and fatigue become more pronounced, but future studies are needed to investigate this hypothesis.

In this study, gait speed and stride length were significantly associated with the MDS‐UPDRS motor score, adjusting for dopaminergic state. This is important since there are very few items related to gait and balance on the MDS‐UPDRS. Thus, the digital insole may facilitate longitudinal within‐individual comparisons in trials of PD progression, dose–response intervention effects, phenotypic comparison studies, and randomized controlled trials, all of which may use clinical motor rating scales as outcome measures.

The results regarding the patient experience survey were positive. Based on the patient feedback, digital insole makers should consider the thickness of the insole, height of the heel, ease of adjustment (trimmable), and provision of different arch supports and sizes to inclusively cater for the wide variety of foot shapes. From a clinical study design perspective, investigators and/or companies may wish to consider measuring or scanning patients' feet to ensure correct size and arch support (if available), allowing time for fitting and adjustment or provision of a fitting service to reduce patient burden and ensure correct fit. Further patient experience research should focus on the assessment of longer‐term comfort using a patient daily diary and assess the impact on comfort and ease of use of digital insoles in other types of footwear.

There are caveats to this study. This study did not assess the validity of gait variability, asymmetry, turning, and base of support (i.e., step width) parameters measured by the digital insole for concordance with the GAITRite walkway or responsiveness to dopaminergic medication (rather than ON/OFF state), or associations with MDS‐UPDRS motor items. Since gait asymmetry is prevalent in patients with PD [30], and gait variability and turning are abnormal and highly associated with fall risk [31, 32, 33, 34], these are important variables to consider in future studies. An abnormal base of support (either reduced or increased) is associated with reduced postural control in patients with PD [35, 36] and other movement disorders, and therefore may be an important outcome measure. This could also be investigated in future validity studies.

The digital insoles and GAITRite systems do not automatically provide the same gait characteristic outputs, and therefore characteristics from some gait domains (including asymmetry, some variability measures, and turning) were not compared. Data was also not synced from the GAITRite with that of the ReGo insole, precluding step‐by‐step comparisons. Instead, the average gait data automatically generated by each system's software was compared. The rationale was that this is how the data would be extracted for clinical trials instead of more complicated step‐by‐step analyses.

It is also important to acknowledge the technological limitations of using pressure insoles to measure gait in PD [37, 38, 39]. These are related to both the device architecture and the practical aspects of their use. Force sensor accuracy may be affected by signal drift during long recording sessions, and the quality of the insole signals, which are sensitive to factors such as sensor placement relative to the foot, type of footwear, walking surface, and calibration procedures. For this reason, maintaining consistent measurement conditions and performing calibration before each recording is recommended, especially when monitoring changes in digital gait measures over time. The ReGo insoles have on‐board battery and data storage, enabling up to approximately 30 h of recording, though actual capacity depends on sampling rate and the test modalities selected within the OpenGo software application.

Our sample size was small and relatively homogeneous, with patients predominantly in Hoehn and Yahr stage 2. While these factors were similar to other studies testing the validity and clinical utility of digital insoles in patients with PD [7, 8, 10], this is a first step in determining feasibility and validity for PD research or other applications. The next steps could include assessment of the digital insoles in a larger sample size that incorporates a broad range of PD phenotypes and disease severity (i.e., those with early disease not on symptomatic treatment, given the heterogeneous nature of PD, or those with freezing of gait and/or requiring the use of an assistive device). Furthermore, we could longitudinally assess whether they might be more sensitive digital measures of disease progression over shorter, clinically relevant, and feasible clinical trial times compared to the reference system MDS‐UPDRS. We could also assess digital insoles in terms of their relatedness to reference system gait and balance performance instruments like the Mini‐Balance Evaluation Systems Test [40] and patient‐reported outcome measures, including the Activities‐Specific Balance Confidence scale [41], a self‐reported scale of balance confidence for performing functional balance and walking tasks which is highly predictive of fall risk in the elderly and in patients with PD [42], and the Neurological Quality of Life scale, which has been validated in patients with PD [43, 44]. Ideally, the digital insole would be added to clinical trials or large observational studies in patients with PD to facilitate the data needed to incorporate it as a clinical trial outcome measure. The ultimate goal is to be able to use this type of wearable device not only as a research outcome measure but also for remote disease monitoring, tracking exercise responsiveness, and determining the effects of interventions to improve quality of life [45] in patients with PD. Additional studies are needed with a broader range of disease severity and to confirm these findings in free‐living settings, which would also require larger sample sizes due to increased variability expected in natural environments.

## Author Contributions

D.A.H., K.K., E.R., B.K., R.B., M.F.W., and O.L. contributed to the conception and design of the study. E.T., N.L.P., N.D., K.K., J.A.O., D.A.H., and R.B. were involved in the collection of data. M.F.W. and R.J.A. contributed statistical/data analysis planning support and P.R., R.B., and R.J.A. performed data cleaning and analysis. D.A.H., K.K., R.J.A., J.A.O., B.K., E.R., R.B., M.F.W., and O.L. contributed to data interpretation. D.A.H., K.K., J.A.O., R.J.A., E.R., S.S., R.B., M.F.W., and O.L. contributed to the writing of the manuscript and all authors reviewed and approved the final manuscript. Administrative, technical, or logistic support/study supervision or coordination was provided by A.‐M.V.‐K. and R.A.

## Funding

This work was supported by Regeneron Pharmaceuticals.

## Conflicts of Interest

D.A.H. has received research funding from NINDS, DOD, ASAP, MJFF, CHDI, Uniqure, Neurocrine, Alterity, Lundbeck, SAGE, and Regeneron. She receives support for editorial work at Neurology Today, Annals of Neurology, and Parkinsonism and Related Disorders. K.K.: There are no conflicts of interest. R.J.A., B.K., E.R., P.R., R.B., D.P., A.‐M.V.‐K., S.S., R.A., M.F.W., and O.L. are employees of and shareholders in Regeneron Pharmaceuticals Inc. E.T.: There are no conflicts of interest. N.L.P.: There are no conflicts of interest. ND: There are no conflicts of interest. J.A.O. has received research funding from the NIH, DOD, VA, and HDSA.

## Supporting information


**Figure S1:** Comparison of gait characteristics to the MDS‐UPDRS motor score.

**Table S1:** Device User Experience Questionnaire: question summary.

## Data Availability

Qualified researchers may request access to study documents (including the clinical study report, study protocol with any amendments, blank case report form, statistical analysis plan) that support the methods and findings reported in this manuscript. Individual anonymized participant data will be considered for sharing (1) once the product and indication has been approved by major health authorities (e.g., FDA, EMA, PMDA, etc.) or development of the product has been discontinued globally for all indications on or after April 2020 and there are no plans for future development (2) if there is legal authority to share the data and (3) there is not a reasonable likelihood of participant reidentification. Submit requests to https://vivli.org/.
