# Supplementary material for: Validity of a Wearable Digital Insole for Assessing Gait ON and OFF in Parkinson's Disease
Source: Ann Clin Transl Neurol. 2026 Feb 24;13(8):1662–72. doi: 10.1002/acn3.70333 (PMC13394956; doi:10.1002/acn3.70333)
Supplement: Supplementary file 2 [file ACN3-13-1662-s002.docx]

**Appendix**

**Supplemental Figure 1. Comparison of gait characteristics to the MDS-UPDRS motor score**

The y-axis contains the standardized association between each gait characteristic and the MDS-UPDRS Part III. All the gait characteristics were scaled and centered; hence, the interpretation is in terms of standard deviation increases of the gait characteristics. The x-axis contains the Cohen’s d of the difference between the ON and OFF medication states. Since the gait characteristics were scaled and centered, the differences are in standard deviation units. Gait characteristics as measured by Moticon (M) are presented in circles and gait characteristics as measured by GAITRite (G) are presented as inverted triangles. Pink data points are gait characteristics that do not have a significant association with the MDS-UPDRS Part III but have a significant mean difference between the ON and OFF medication states. Blue data points are gait characteristics that have a significant association with the MDS-UPDRS Part III and a significant mean difference between the ON and OFF medication states.

MDS-UPDRS, Movement Disorders Society’s Unified Parkinson’s Disease Rating Scale.
