# Supplementary material for: Validity of a Wearable Digital Insole for Assessing Gait ON and OFF in Parkinson's Disease
Source: Ann Clin Transl Neurol. 2026 Feb 24:10.1002/acn3.70333. Online ahead of print. doi: 10.1002/acn3.70333 (PMC13394956; doi:10.1002/acn3.70333)
Supplement: Supplementary file 2 — Table S1: Device User Experience Questionnaire: question summary. [file ACN3-9999-0-s001.docx]

**Supplemental Table 1**

**Device User Experience Questionnaire: Question Summary**

| No. | Question | Responses |
| --- | --- | --- |
| 1 | What did you like most about wearing the insoles? | Open |
| 2 | What did you not like, if anything, about wearing the insoles? | Open |
| 3 | What extent were you comfortable wearing the insoles?  Please select one response. | Very comfortable  Comfortable  Neither comfortable or uncomfortable  Uncomfortable  Very uncomfortable |
| 4 | To what extent was it easy to use the insoles?  Please select one response. | Very easy  Easy  Neither easy or difficult  Difficult  Very difficult |
| 5 | Did the insoles require to adjust while completing the task? | Yes  No |
| 6 | If Q.5 is answered as Yes, to what extent was it easy to adjust the insoles?  Please select one response. | Very easy  Easy  Neither easy or difficult  Difficult  Very difficult |
| 7 | To what extent were you satisfied with the insoles? Please select one response. | Very satisfied  Satisfied  Neither satisfied or dissatisfied  Dissatisfied  Very dissatisfied |
| 8 | To what extent would you be willing to use the insoles at your home to complete the walking task more frequently, as a part of clinical trial? | Very willing  Willing  Neutral  Unwilling  Very unwilling |
| 9 | If Q.8 is answered as Willing or Very willing, please select one response, corresponding to the most frequent assessment that you would find acceptable. | Daily  Weekly  Bi-Weekly  Monthly  Other (i.e. less frequent than monthly). Please elaborate _________________ |
| 10 | Did the insoles cause discomfort or any irritation while completing the task? | Yes  No |
| 11 | Did the insoles overheat while completing the task? | Yes  No |
| 12 | To what extent would you be recommending insoles to others? | Very likely  Likely  Neither likely nor unlikely  Unlikely  Very unlikely |
|  | **Thank you for completing this questionnaire.** |  |
